# Does inhalation injury predict mortality in burns patients or require redefinition?

**DOI:** 10.1371/journal.pone.0185195

**Published:** 2017-09-27

**Authors:** Youngmin Kim, Dohern Kym, Jun Hur, Jaechul Yoon, Haejun Yim, Yong Suk Cho, Wook Chun

**Affiliations:** Department of Surgery and Critical Care, Burn Center, Hangang Sacred Heart Hospital, Hallym University Medical Center, Seoul, Korea; National Yang-Ming University, TAIWAN

## Abstract

Inhalation injury is known to be an important factor in predicting mortality in burns patients. However, the diagnosis is complicated by the heterogeneous presentation and inability to determine the severity of inhalation injury. The purpose of this study was to identify clinical features of inhalation injury that affect mortality and the values that could predict the outcome more precisely in burns patients with inhalation injury. This retrospective observational study included 676 burns patients who were over 18 years of age and hospitalized in the Burns Intensive Care Unit between January 2012 and December 2015. We analyzed variables that are already known to be prognostic factors (age, percentage of total body surface area (%TBSA) burned, and inhalation injury) and factors associated with inhalation injury (carboxyhemoglobin and PaO_2_/FiO_2_ [PF] ratio) by univariate and multivariate logistic regression. Age group (odds ratio [OR] 1.069, *p*<0.001), %TBSA burned (OR 1.100, *p*<0.001), and mechanical ventilation (OR 3.774, *p*<0.001) were identified to be significant predictive factors. The findings for presence of inhalation injury, PF ratio, and carboxyhemoglobin were not statistically significant in multivariate logistic regression. Being in the upper inhalation group, the lower inhalation group, and having a PF ratio <100 were identified to be significant predictors only in univariate logistic regression analysis (OR 4.438, *p*<0.001; OR 2.379, *p*<0.001; and OR 2.765, *p*<0.001, respectively). History and physical findings are not appropriate for diagnosis of inhalation injury and do not predict mortality. Mechanical ventilation should be recognized as a risk factor for mortality in burns patients with inhalation injury.

## Introduction

Inhalation injury is defined as the presence of airway or lung damage caused by heat or the chemical products of combustion. Inhalation injury is the third most important factor after age and total body surface area (TBSA) burned in predicting the mortality rate of burns patients [1]. Inhalation injury causes decreased oxygen perfusion because of direct heat injury to the upper respiratory tract, chemical stimulation of the lower respiratory tract, and injury in response to noxious gases, including carbon monoxide and cyanide, and occurs in up to one third of patients with major burns [2]. Despite advances in critical care, the mortality rate in burns patients with inhalation injury is reported to be about 10%–30% and increases with increasing TBSA burned [3, 4]. Accurate diagnosis and immediate treatment of inhalation injury are known to be critical for better outcomes, given that the inhalation injury has a close relationship with secondary pneumonia, mechanical ventilation, and acute respiratory distress syndrome. Classically, the diagnosis of inhalation injury relies primarily on a history of smoke exposure in confined spaces and prolonged rescue, and/or physical findings such as scorched nasal hair, carbonaceous sputum in the oropharynx, facial burns, and voice changes [4–6]. These findings can be diagnosed by fiberoptic bronchoscopy [4, 7]. However, this method of diagnosis is complicated by the heterogeneous presentation and inability to determine the severity of inhalation injury. The purpose of this study was to identify clinical features of inhalation injury that affect mortality and the values that could predict the outcome more precisely in burns patients with inhalation injury.

## Methods

This retrospective observational study included 676 burns patients who were older than 18 years and admitted within 24 hours of a burn injury to the Burns Intensive Care Unit (BICU) at Hangang Sacred Heart Hospital, Hallym University Medical Center, Seoul, Korea, between January 2012 and December 2015. The study was approved by the Institutional Review Board of Hangang Sacred Heart Hospital. The need for informed consent was waived because of the retrospective nature of the study. We recorded the following variables for each patient: age, sex, TBSA burned, presence of full-thickness burns, length of stay in hospital, and length of stay in the intensive care unit. The Acute Physiology and Chronic Health Evaluation (APACHE) III score and the Abbreviated Burn Severity Index (ABSI) score [8], which was calculated by assigning a numeric value to the parameters of age, sex, extent of burns, presence of full-thickness burns, and presence of inhalation injury according to their severity and all of five values were then summed, were also recorded. The extent of burns was measured by an experienced burns surgeon using a modified Lund and Browder chart [9]. Carboxyhemoglobin level and the PaO_2_/FiO_2_ (PF) ratio are known indicators of the severity of inhalation injury and were measured by arterial blood gas analysis on admission.

The 676 patients were divided into four groups (normal, subjective, upper, and lower) according to inhalation status. The subjective group included patients with only a history of smoke exposure in a confined space and prolonged rescue, and/or physical findings including singed facial hair, carbonaceous sputum in the oropharynx, facial burns, and voice change. Patients who showed any degree of compromised airway obstruction on laryngoscopy and were intubated were divided into upper and lower inhalation groups; patients identified on bronchoscopy to have any injuries such as edema, carbonaceous material, blistering, inflammation, and ulceration below the level of the trachea were included in the lower group. Intubated patients who did not undergo bronchoscopy because lower airway inhalation injury was not suspected by the attending physician and those for whom findings on bronchoscopy were normal were included in the upper group.

The ventilation group included patients who commenced mechanical ventilation within 48 hours of their injury to evaluate if ventilation affects mortality. Mechanical ventilation was applied in patients with a sustained respiratory rate greater than 35 and a subjective sense of fatigue. Initial ventilator settings were a ventilator rate of 12–18 breaths per minute, a tidal volume of 8 mL/kg, an inspiratory to expiratory ratio of 1:3–1:2, a flow rate of 40–60 L/min, and the lowest possible FiO_2_ to maintain an oxygen saturation of greater than 90%. We also divided the patients into four groups depending on the PF ratio (>300, 200–300, 100–200, and <100).

All continuous variables are presented as the mean ± standard deviation, and the frequencies of categorical variables are presented as percentages. Continuous variables were analyzed using the independent *t*-test when their distribution was normal and the Mann-Whitney *U* test when the distribution was not normal. Categorical variables were analyzed using the chi-square test. Multivariate logistic regression analysis was performed to evaluate whether inhalation injury and the PF ratio (both independent variables that were categorized according to group) are risk factors for burns-related mortality and inhalation injury. The effects of these two parameters were compared by Kaplan-Meier survival curve analysis with the log-rank test. A probability value of ≤0.05 was considered to be statistically significant. The statistical analysis was performed using IBM SPSS Statistics version 24 software (IBM Corp., Armonk, NY, USA).

## Results

### Patient demographic characteristics and comparison between survivors and non-survivors

Two hundred and ninety-seven of the 676 patients admitted to the Burns Intensive Care Unit were diagnosed to have inhalation injury, giving an overall incidence of 43.9%. One hundred and eighteen patients (39.7%) with inhalation injury were in the subjective group, 81 (27.3%) were in the upper group, and 98 (33.0%) were in the lower group. Bronchoscopy was performed in 131 patients (98 in the upper group and 33 in the lower group). Bronchoscopy was not performed in 48 patients (59.3%) in the upper group because the possibility of lower airway injury was considered low by the attending physician. Two hundred and seventy-four patients underwent mechanical ventilation within 2 days of admission. The mortality rate was 25.6% (n = 173/676). The mean patient age was 48.9 years. A male predominance was noted (male to female ratio 4.5:1). The mean %TBSA burned was 36.8% overall and differed significantly between survivors (26.7%) and non-survivors (66.2%). Five hundred and forty-one (80.0%) of the 676 patients had full-thickness burns. Mean Abbreviated Burn Severity Index and APACHE II scores were significantly higher in non-survivors than in survivors (11.89 vs 59.6). The mean carboxyhemoglobin level was 2.16%, with no significant difference between survivors and non-survivors. The mean PF ratio was 235.7 overall, and was significant higher in survivors than in non-survivors (248.0 vs 200.0; Table 1).

**Table 1:** Patient demographics and comparisons between survivors and non-survivors.

	Total(n = 676)	Survivors(n = 503)	Non-survivors(n = 173)	*p*-value
Mean age (years)	48.9 ± 14.8	47.1 ± 13.9	54.2 ± 16.0	<0.001
Sex (male:female)	553:123	414:89	139:34	0.569
Mean % TBSA burned	36.8 ± 26.0	26.7 ± 17.5	66.2 ± 24.2	<0.001
Full-thickness burns, n (%)	541 (80.0%)	379 (75.3%)	162 (93.6%)	<0.001
ABSI score	8.49 ± 2.96	7.32 ± 2.09	11.89 ± 2.44	<0.001
APACHE III	36.5 ± 21.5	28.6 ± 16.0	59.6 ± 18.9	<0.001
LOS in ICU (days)	20.0 ± 23.9	21.8 ± 25.5	14.6 ± 17.2	<0.001
LOS in hospital (days)	48.3 ± 36.5	59.9 ± 34.1	14.6 ± 17.2	<0.001
Carboxyhemoglobin level (%)	2.16 ± 3.85	2.03 ± 3.62	2.52 ± 4.42	0.169
PF ratio	235.7 ± 130.7	248.0 ± 132.2	200.0 ± 119.9	<0.001
Mechanical Ventilation	274(40.5%*)	131(26.0%)	143(82.7%)	<0.001
Inhalation injury				<0.001
Normal	379(56.1%*)	302(79.7%)	77(20.3%)	
Subjective	118 (17.5%*)	102 (86.4%)	16 (13.6%)	
Upper	81 (12.0%*)	38 (46.9%)	43 (53.1%)	
Lower	98 (14.5%*)	61 (62.2%)	37 (37.8%)	
PF ratio				<0.001
>300 (normal)	162 (24.0%*)	124 (24.7%)	38 (22.0%)	
200–300	235 (34.8%*)	190 (37.8%)	45 (26.0%)	
100–200	170 (25.1%*)	130 (25.8%)	40 (23.1%)	
<100	109 (16.1%*)	59 (11.7%)	50 (28.9%)	

ABSI, Abbreviated Burn Severity Index; APACHE, Acute Physiology and Chronic Health Evaluation Score; ICU, intensive care unit; LOS, length of stay; PF ratio, ratio of arterial O_2_ pressure to fraction of inspired oxygen; TBSA, total body surface area,

* The percentage within total group

### Univariate and multivariate logistic regression analyses and analysis of survival according to parameters associated with pulmonary function

**Fig 1:**
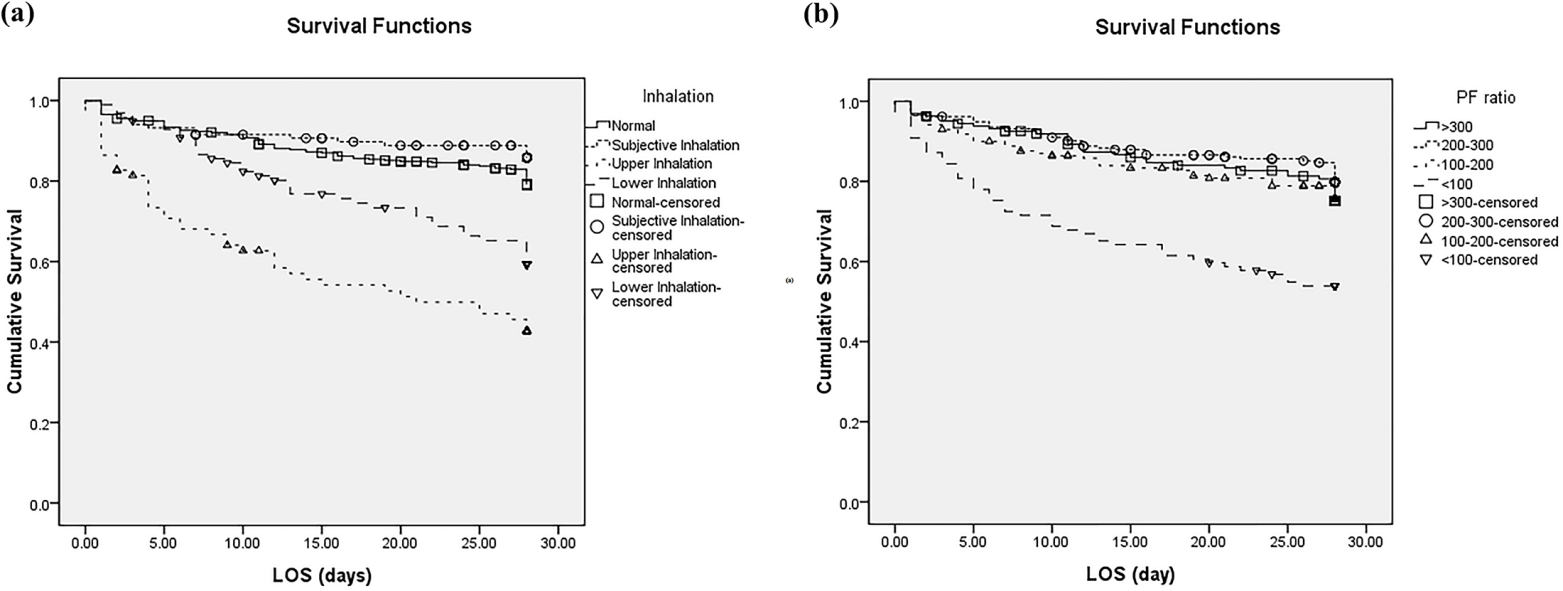
Analysis of survival according to inhalation injury (a) and PF ratio (b). Significant survival differences by inhalation injury (*p*<0.001) and PF ratio (*p*<0.001) are seen. PF ratio, ratio of arterial O_2_ pressure to fraction of inspired oxygen; LOS, length of stay in hospital.

We analyzed the variables already known to be prognostic factors (age, %TBSA burned, inhalation injury, and mechanical ventilation) and factors associated with inhalation injury (carboxyhemoglobin and PF ratio) by univariate and multivariate logistic regression. Age group (odds ratio [OR] 1.069, *p*<0.001), %TBSA burned (OR 1.100, *p*<0.001), and mechanical ventilation (OR 3.774, *p*<0.042) were identified to be significant predictive factors. The findings for inhalation injury, PF ratio, and carboxyhemoglobin were not statistically significant in multivariate logistic regression analysis. A PF ratio <100 and being in the upper or lower inhalation group were identified to be significant predictors only in univariate logistic regression analysis (OR 2.765, *p*<0.001; OR 4.438, *p*<0.001; and OR 2.379, *p*<0.001, respectively; Table 2). A 28 day-survival analysis revealed a statistically significant difference in survival according to inhalation injury (*p*<0.001) and PF ratio (*p*<0.001; Fig 1).

**Table 2:** Univariate and multivariate logistic regression analysis of potential predictors of mortality, including patient age, %TBSA burned, inhalation injury, PF ratio, and carboxyhemoglobin level at presentation.

	Univariate		Multivariate	
	OR (95% CI)	*p*-value	OR (95% CI)	*p*-value
Age	1.034 (1.021–1.046)	<0.001	1.069 (1.046–1.092)	<0.001
% TBSA burned	1.083 (1.070–1.096)	<0.001	1.100 (1.081–1.119)	<0.001
Mechanical Ventilation	5.494 (3.661–8.244)	<0.001	3.774 (1.051–13.552)	0.042
Inhalation injury		<0.001		0.107
Subjective	0.615 (0.343–1.103)	0.103	0.467 (0.191–1.143)	0.095
Upper	4.438 (2.684–7.339)	<0.001	1.907 (0.590–6.164)	0.281
Lower	2.379 (1.474–3.841)	<0.001	0.775 (0.201–2.995)	0.712
PF ratio		<0.001		0.180
200–300	0.773 (0.475–1.258)	0.300	0.622 (0.277–1.394)	0.249
100–200	1.004 (0.604–1.668)	0.988	1.334 (0.586–3.034)	0.493
<100	2.765 (1.638–4.668)	<0.001	1.428 (0.590–3.455)	0.430
Carboxyhemoglobin level	1.031 (0.986–1.077)	0.178	1.081 (0.987–1.184)	0.093

CI, confidence interval; OR, odds ratio; PF ratio, ratio of arterial O_2_ pressure to fraction of inspired oxygen; TBSA, total body surface area

## Discussion

In a meta-analysis of prognostic factors in burns patients with inhalation injury, Colohan et al [10] reported that the mortality rate (27.6%) doubled in burns patients with inhalation injury and that the TBSA burned and patient age were also predictors of mortality. A similar mortality rate (25.6%) was documented in the present study. The incidence of inhalation injury in our study was 43.9%, which is higher than the incidence rate of 10%–20% reported in other studies [4–7]. We believe that the likely reason for this is that most burns patients suspected to have inhalation injury in South Korea are transferred to our center because it is the only tertiary referral burns center in the region. Hassan et al [11] identified inhalation injury, TBSA burned, patient age, and the PF ratio to be predictors of mortality. Although inhalation injury is known to be an important predictor of mortality in burns patients, there is still no clear standard for diagnosis and no factors reflecting its severity have been identified. In the present study, inhalation injury was not associated with history, physical findings, or intubation, but was associated with mechanical ventilation. Similarly, the carboxyhemoglobin level and PF ratio did not reflect the severity of inhalation injury. It seems unlikely that the history, clinical findings, or intubation per se are absolute indicators of inhalation injury, given the lack of reliability of these factors when used as criteria for inhalation injury. The unreliability of physical findings can be explained by the two (chemical and thermal) mechanisms of inhalation injury [4]. Chemical injury is caused by inhalation of toxic gases, which causes problems in the tracheobronchial tree, i.e., the lower airway [5], whereas in the case of thermal injury, the hot air rarely progresses to the lower airway because of cooling in the pharynx [12]. Upper airway damage caused by thermal injury can be confirmed by laryngoscopy and lower airway damage can be confirmed by bronchoscopy. Therefore, it is necessary to confirm lower airway damage by bronchoscopy because physical findings, such as scorched nasal hair, carbonaceous sputum in the oropharynx, and facial burns, are indicators of thermal injury at the level of the pharynx and cannot predict lower airway damage.

In our study, because chemical injury could not be confirmed by direct observation, we analyzed the carboxyhemoglobin level and PF ratio. The carboxyhemoglobin level was not a statistically significant factor in univariate or multivariate regression analysis. Traditionally, the carboxyhemoglobin level has been considered to be an indicator of inhalation injury; however, its utility is in fact limited because of its short half-life of 3–4 hours, which becomes even shorter during oxygenation [3]. Endorf et al [7] reported the PF ratio to be a significant predictor of mortality in burns patients with inhalation injury. Our results showed that a PF ratio <100 was a statistically significant factor in univariate regression but not in multivariate regression analysis. Therefore, we believe that a PF ratio <100 in burns patients with inhalation injury might be an important prognostic indicator.

Mechanical ventilation was identified to be a significant predictor of mortality. We believe that our mechanical ventilation group included patients with chemical damage to the lower airway and lung parenchyma as well as thermal damage to the upper airway, who were at high risk for lung complications, including secondary pneumonia and acute respiratory distress syndrome. In our study, mechanical ventilation was not performed in the normal group or the subjective group but was performed in 48 patients (59.3%) in the upper inhalation injury group and 84 (85.7%) in the lower inhalation injury group (p<0.001). Therefore, we can infer that the presence of inhalation injury would affect whether mechanical ventilation is performed or not. Mechanical ventilation is predictive of the fluid volume administered during the first 24 hours and is associated with fluid accumulation in burns patients [13, 14]. Recognition of ventilator-associated lung injury (VALI) is now increasing. The repeated stress of mechanical ventilation on the small airways and alveoli causes the inflammatory process that occurs in VALI [15]. For this reason, we consider mechanical ventilation to be a risk factor and avoid using it unless necessary.

The present study has several limitations. First, it had a retrospective single-center design, which is well known to be associated with a risk of bias. However, many patients with severe burns in Korea are transferred to our center because it is the only university-affiliated burns center in the region and has been designated as “The Emergency Center for Burn Care” by the Korean Ministry of Health, Welfare, and Family Affairs. Therefore, it is possible that our findings are representative of all burns cases in Korea. Second, bronchoscopy has not been performed routinely at our center when inhalation injury is suspected. Currently, inhalation injury is confirmed by bronchoscopy [6]. However, we confirm inhalation injury by bronchoscopy only if lower airway injury is suspected or if needed for treatment purposes, so there was a degree of selection bias in both the upper and lower groups. Third, we do not use any bronchoscopic grading system for inhalation injury, such as the Abbreviated Injury Score (AIS) [7]. Bronchoscopic grading has been reported to be associated with and predictive of increased morbidity and mortality [16]. In this study, if there were any abnormality in the lower airway below the level of the trachea on bronchoscopy, lower inhalation injury was assumed but the severity was not assessed. Therefore, a further study should be performed prospectively to confirm the indicators of inhalation injury in burns patients using bronchcoscopy and a grading system.

## Conclusion

Inhalation injury has been used as a predictor of clinical outcome, but there is no standard definition or diagnostic pathway for clinicians to follow. The results of this study show that history and physical findings for inhalation injury do not predict mortality or level of inhalation injury and that the PF ratio is not a statistically significant predictor of mortality. Thus, there are indicators of the risk of inhalation injury and factors that could predict mortality. Therefore, redefinition of inhalation is necessary, and mechanical ventilation needs to be recognized as one of the risk factors for mortality in burns patients with inhalation injury.

## Supporting information

S1 Dataset Datasets of this study. (XLSX)
